# Effects of the HDAC6/8 Inhibitor MC1568, Alone and in Combination With Fluconazole, in Non‐*Albicans Candida* Species Infections

**DOI:** 10.1002/mbo3.70188

**Published:** 2026-07-07

**Authors:** Andrea Giammarino, Chiara Lambona, Alessia Raucci, Giovanna Simonetti, Salvatore Macrì, Angela Iuzzolino, Daniela Trisciuoglio, Sergio Valente, Gustavo Giusiano, Letizia Angiolella, Antonello Mai

**Affiliations:** ^1^ Department of Public Health and Infectious Diseases Sapienza University of Rome Rome Italy; ^2^ Department of Drug Chemistry and Technologies Sapienza University of Rome Rome Italy; ^3^ IBPM Institute of Molecular Biology and Pathology CNR National Research Council of Italy Rome Italy; ^4^ Departamento de Micología, Instituto de Medicina Regional Universidad Nacional del Nordeste Argentina

**Keywords:** adherence, biofilm, *Candida* spp, drug combination, *Galleria mellonella* model, histone deacetylases, lysine deacetylation

## Abstract

Fungal infections pose a severe health risk, particularly in immunocompromised individuals, with *Candida* species being a leading cause of bloodstream infections. The rise of non‐*albicans Candida* (NAC) infections, accounting for 35%–65% of candidemia cases, underscores the urgent need for novel antifungal strategies. Current treatments, including echinocandins and azoles, are increasingly challenged by drug‐resistant strains. This study evaluated the antifungal efficacy of three clinically approved histone deacetylase inhibitors (HDACi)—Vorinostat, Romidepsin, and Tucidinostat—along with the HDAC6/8‐selective inhibitor MC1568, alone and in combination with Fluconazole. The Fluconazole/MC1568 combination exhibited significant synergistic effects, reducing biofilm formation and enhancing antifungal activity, particularly against *C. guillermondii*, *C. lusitaniae*, and *C. tropicalis*. In *Galleria mellonella* larvae, this combination increased survival by 70% in *C. tropicalis* infections. When tested in human retinal pigment epithelium (RPE) cells, three different MC1568/Fluconazole combinations displayed low toxicity. These findings highlight the potential of the HDAC6/8 inhibitor MC1568 as adjunct therapy to combat NAC infections, warranting further research to optimize antifungal efficacy while minimizing toxicity.

## Graphical abstract

This study explores new antifungal strategies to combat non‐*albicans Candida* species. We tested clinically approved HDAC inhibitors—Vorinostat, Romidepsin, Tucidinostat—and the HDAC6/8‐selective MC1568, alone and with Fluconazole. The Fluconazole/MC1568 combination significantly enhanced antifungal activity and reduced biofilm formation. In an in vivo model, this combo improved survival by 70% in *C. tropicalis* infections. These results support further investigation of MC1568 as a promising adjunct antifungal therapy.

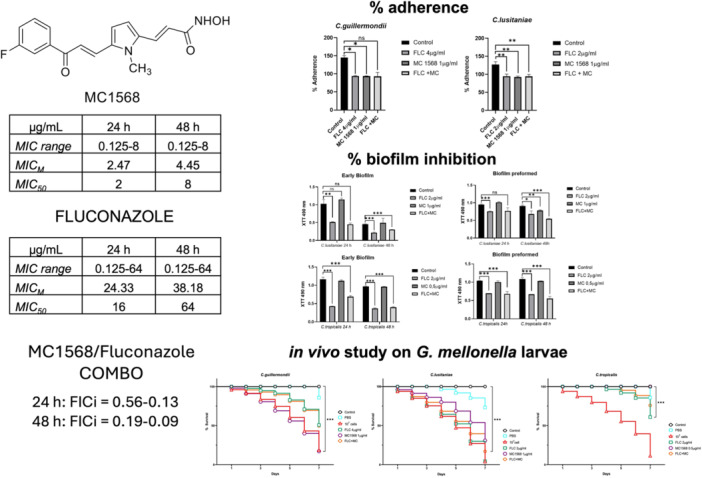

## 1 Introduction

*Candida* spp. comprises a group of yeasts and dimorphic fungi that are commonly found in the environment, as well as on human skin and mucous membranes (Talapko et al. 2021). Although generally harmless under normal conditions, they can become opportunistic pathogens, particularly in immunocompromised individuals. Specifically, *Candida* spp. can cause a spectrum of diseases, ranging from superficial mucocutaneous infections (such as oral thrush and vaginitis) to life‐threatening invasive candidiasis (systemic and deep‐seated infections) (Stringaro et al. 2022).

Among *Candida* species, the prevalence of non‐*albicans Candida* (NAC) species is increasing, now accounting for 35%–65% of all candidemia cases. The most common NAC species include *C. parapsilosis* (20%–40% of all *Candida* infections), *C. tropicalis* (10%–30%), *C. krusei* (10%–35%) *C. glabrata* (5%–40%), and the emerging *C. auris* (4% of *Candida* infections, but with 30%–60% of mortality) (Eix and Nett 2025). In addition, two other species, *C. lusitaniae* and *C. guilliermondii*, are responsible for 2%–8% and 1%–5% of infections, respectively (Krcmery and Barnes 2002).

The pathogenicity of *Candida* spp. is influenced by various factors, including the virulence of the strain, the immune status of the host, and the local environment (Mba and Nweze 2020). These fungi produce virulent factors such as adhesins, which mediate attachment to host cells and surfaces. Adhesion is the initial step in fungal colonization and plays a crucial role in persistence and dissemination within the host (Ghannoum et al. 1991). Similarly, biofilm formation is a key virulence factor that enhances fungal pathogenesis (Silva et al. 2009). *Candida* species form complex, multicellular biofilms that facilitate attachment to host tissues, catheters, implants, and medical devices. Biofilm development occurs over 38–72 h in multiple stages: initial adhesion to biotic or abiotic surfaces, proliferation and filamentation of adhered cells, maturation, and eventual dispersion, in which yeast cells detach and spread. The biofilm formation process varies among *Candida* species (Malinovska et al. 2023).

Some *Candida* species exhibit intrinsic or acquired resistance to antifungal agents (Miceli et al. 2011; Vitali et al. 2017). Antifungal susceptibility varies significantly: Fluconazole resistance has been reported in approximately 75% of *C. krusei* isolates, 35% of *C. glabrata*, and 10%–25% of *C. tropicalis* and *C. lusitaniae* (Krcmery and Barnes 2002). The increasing prevalence of drug‐resistant *Candida* strains has driven efforts to better understand the mechanisms underlying biofilm‐associated antifungal tolerance and to develop new therapeutic strategies.

Histone deacetylases (HDACs) are enzymes that remove acetyl groups from lysine residues on histones and other cellular proteins (Bondarev et al. 2021; Glozak et al. 2005), influencing diverse biological processes such as signal transduction, cell growth, apoptosis, human diseases, cancer, and infections caused by eukaryotic microorganisms (Fioravanti et al. 2020; Yoshida et al. 2017). HDACs are classified into four groups: class I (HDAC1‐3, ‐8), class IIa (HDAC4, ‐5, ‐7, ‐9), class IIb (HDAC6, ‐10), and class IV (HDAC11) (Witt et al. 2009).

HDAC inhibitors (HDACi) belong to various chemical classes and function by binding to the catalytic site of HDACs, inducing cell cycle arrest, apoptosis, and differentiation (Di Bello et al. 2022; Raucci et al. 2024). Protein acetylation is a widespread regulatory modification in fungi, influencing numerous cellular processes and the overall cell cycle (Etier et al. 2022; Zhang et al. 2024). Consequently, HDACi have emerged as promising antifungal agents. Developing HDACi that selectively target fungal‐specific HDACs with low effects in mammalian cells could reduce toxicity and provide novel therapeutic options (Pang et al. 2022). While the effects of HDACi in *C. albicans* have been widely studied (Garnaud et al. 2016; Smith and Edlind 2002), data on NAC species remain limited.

In 2002, Smith and Edlind demonstrated that Trichostatin A, a pan‐HDACi, significantly downregulated *Erg11* and *Cdr* genes in *C. albicans* and other *Candida* spp. following exposure to sterol biosynthesis inhibitors such as Fluconazole and Terbinafine (Smith and Edlind 2002). The *Erg11* gene produces the lanosterol 14α‐demethylase enzyme, vital for synthetizing ergosterol in fungi. Azole antifungal drugs, for example, Fluconazole, block the ergosterol biosynthesis pathway, making this the primary target. Numerous molecular mechanisms can lead to azole resistance, e.g. alteration and/or overexpression of *Erg11*, and upregulation of efflux transporter genes, such as *Mdr1* and *Cdr1* genes (El‐Kholy et al. 2023). Vorinostat (SAHA, suberoylanilide hydroxamic acid) (Richon et al. 1996), the first synthetic hydroxamate HDACi approved by the U.S. Food and Drug Administration (FDA) in 2006 for advanced primary cutaneous T‐cell lymphoma (Mann et al. 2007) and currently in clinical trials—alone or in combination with other chemotherapeutics—for a number of tumors (El Omari et al. 2025), was later shown to inhibit Fluconazole‐induced resistance in *Candida* cultures (Mai et al. 2007). Additionally, Vorinostat reduced *C. albicans* adherence to human pneumocytes by 90% and inhibited serum‐induced germination (Simonetti et al. 2007).

A recent study by de Oliveira et al. (2023) reported that SAHA, although not synergistic with azole antifungals, reduced biofilm formation by about 35% in *C. albicans* at 32 μg/mL and by 30% in *C. neoformans* at 64 μg/mL when used alone (de Oliveira et al. 2023). The antifungal drug MGCD290, a Hos2 fungal HDACi, enhances the activity of echinocandins against echinocandin‐resistant *Candida* spp. (Pfaller et al. 2015). Other HDACi, including Romidepsin and Tucidinostat, have been clinically approved for oncological applications (Di Bello et al. 2022). Romidepsin received FDA approval in 2009 for treating peripheral T‐cell lymphoma (PTCL) (Bertino and Otterson 2011). Tucidinostat was approved by the Chinese FDA in 2021 for relapsed or refractory (R/R) PTCL, advanced breast cancer, and R/R adult T‐cell leukemia‐lymphoma (Sun et al. 2022). MC1568 is a selective HDAC6/8 inhibitor with reduced activity against class II HDACs and no activity against HDAC1‐3 (Mai et al. 2005; Noce et al. 2023; Panella et al. 2016). However, the antifungal effects of Romidepsin, Tucidinostat, and MC1568 remain unexplored.

**Figure 1:**
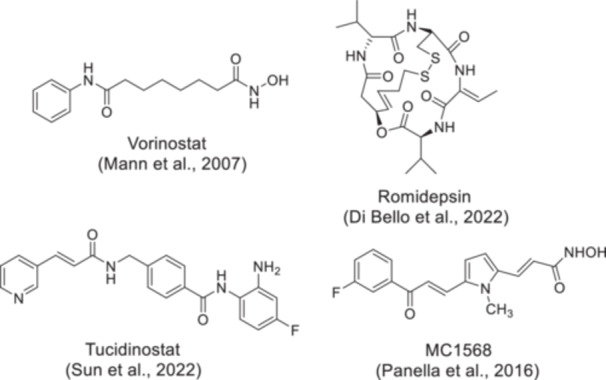
Chemical structures of the HDACi used in this study.

This study evaluates the antimicrobial activity of three clinically approved HDACi—Vorinostat, Romidepsin, and Tucidinostat—along with MC1568 (Figure 1) in NAC isolates. These compounds were selected based on their varying degrees of HDAC selectivity: Vorinostat (pan‐HDACi), Romidepsin (HDAC1‐3/10/11‐selective inhibitor), Tucidinostat (class I/HDAC10/11‐selective inhibitor), and MC1568 (HDAC6/8‐selective inhibitor) (Table 1). The study further investigates the effects of MC1568, alone and in combination with Fluconazole, on fungal adherence and biofilm formation, as well as its in vivo efficacy in *Galleria mellonella* larvae.

**Table 1:** Inhibitory activity of vorinostat, romidepsin, tucidinostat, and MC1568 against the 11 HDAC isoforms (Bondarev et al. 2021; Panella et al. 2016).

HDAC isoform	IC_50_, μM			
	Vorinostat	Romidepsin	Tucidinostat	MC1568
HDAC1	0.060	0.001	0.100	> 300
HDAC2	0.042	0.001	0.200	> 300
HDAC3	0.036	0.001	0.100	> 300
HDAC4	0.020	0.647	> 10	48.2
HDAC5	0.036	> 1	> 10	25.3
HDAC6	0.029	0.226	> 10	5.94
HDAC7	0.129	> 1	> 10	
HDAC8	0.173	> 1	0.700	1.81
HDAC9	0.049	> 1	> 10	
HDAC10	0.060	0.001	0.100	
HDAC11	0.031	0.0003	0.400	

## 2 Materials and Methods

### 2.1 Compounds

The three clinically approved HDACi Vorinostat, Romidepsin, and Tucidinostat, were purchased from MedChemExpress, New Jersey (USA), or BLD‐Pharma, Kaiserslautern (Germany), and were of the highest purity. MC1568 ((*E*)‐3‐(5‐((*E*)‐3‐(3‐fluorophenyl)‐3‐oxoprop‐1‐en‐1‐yl)‐1‐methyl‐1*H*‐pyrrol‐2‐yl)‐*N*‐hydroxyacrylamide) was synthesized in our lab as previously described (Mai et al. 2005; Panella et al. 2016). Briefly, ethyl (*E*)‐3‐(5‐formyl‐1‐methyl‐1*H*‐pyrrol‐2‐yl)acrylate, prepared through Vilsmeyer‐Haack reaction performed on (*E*)‐ethyl 3‐(1‐methyl‐1*H*‐pyrrol‐2‐yl)acrylate (Mai et al. 2003) by using oxalyl chloride and *N*,*N*‐dimethylformamide at 0°C in dichloromethane, was treated with 3‐fluoroacetophenone using sodium ethoxide in ethanol to afford the corresponding ethyl (*E*)‐3‐(5‐((*E*)‐3‐(3‐fluorophenyl)‐3‐oxoprop‐1‐en‐1‐yl)‐1‐methyl‐1*H*‐pyrrol‐2‐yl)acrylate. Such ester was directly converted into hydroxamate MC1568 by reaction with hydroxylamine and potassium hydroxide in ethanol.

### 2.2 Microorganisms and Growth Conditions

This study utilized two blood isolates of *C. haemulonii* (IMR‐M‐L 1293 and IMR‐M‐L1375), two blood isolates of *C. lusitaniae* (IMR‐M‐L 301 and IMR‐M‐L 1112), and two blood isolates of *C. guillermondii* (IMR‐M‐L 1511 and IMR‐M‐L 1444). Additionally, four blood isolates of *C. glabrata* (IMR‐ML 23, IMR‐M‐L 1099, DSY 562, and 43976), two urine isolates of *C. krusei* (IMR‐M‐L 1465 and IMR‐M‐L 4495), and one urine isolate of *C. tropicalis* (14615) were included. Blood isolates and urine isolates were taken from hospitalized men and women by the same percentage, and they were isolated from adult subjects except for *C. lusitaniae* IMR 301, which was isolated from a 3‐year‐old girl. This study was approved by the Ethics and Research Committee of the Instituto de Medicina Regional, Universidad National del Nordeste, Argentina (Renis CE000326). Microorganisms were isolated from blood according to protocol M47‐A CLSI (Principles and Procedures for Blood Cultures, 2nd ed. CLSI Clinical and Laboratory Standards Institute, 2022) and for urine according to protocol M54‐A (Principles and Procedures for Detection and Culture of Fungi in Clinical Specimens. 2nd ed. CLSI guideline). All isolates were identified using the Vitek matrix‐assisted laser desorption ionization–time of flight mass spectrometry (MALDI‐TOF MS) system (Bruker Daltoniks, Bremen, Germany) and deposited in the culture collection (IMR‐M‐L) of the Mycology Department, Instituto de Medicina Regional, Universidad Nacional del Nordeste, Resistencia (Argentina). Before virulence factor analysis, all yeast isolates were grown on Sabouraud Dextrose (SD) agar.

### 2.3 Antifungal Activity of Fluconazole, Vorinostat, Romidepsin, Tucidinostat, and MC1568

The antimicrobial activity of Fluconazole and HDACi against all NAC clinical strains was evaluated using the broth microdilution method, following the CLSI M27‐A4 reference methodology (Alexander 2020). Interpretative breakpoints were applied according to CLSI M27M44S guidelines (Procop 2022b). For *C. lusitaniae* (Procop 2022a), Fluconazole epidemiological cut‐off points were used instead of clinical breakpoints (Angiolella et al. 2024).

### 2.4 Synergism Studies

The checkerboard test (Orhan et al. 2005; White et al. 1996) was performed to assess synergistic interactions between Fluconazole and the tested HDACi. Twelve serial two‐fold dilutions of Fluconazole and each HDACi were prepared, following the broth dilution method used to determine minimum inhibitory concentrations (MICs). Fluconazole dilutions ranged from 0.12 to 128 µg/mL, while HDACi dilutions ranged from 0.12 to 16 µg/mL. Each Fluconazole dilution was mixed with the corresponding HDACi concentration to generate a series of different combinations. *Candida* suspensions (2.5 × 10^3^ yeasts in RPMI 1640) were incubated at 28°C for 24 h. The synergistic effect was determined by calculating the fractional inhibitory concentration index (FICi) (White et al. 1996) using the equation:

$$\mathrm{FICi}=\frac{a}{\mathrm{MICa}}+\frac{b}{\mathrm{MICb}},$$

where *a* and *b* represent the MIC of each substance in combination, and MICa and MICb represent the MIC of each substance individually. The FICi values were interpreted as follows: FICi ≤ 0.5 = Synergism; 0.5 < FICi ≤ 1 = Additivity; 1 < FICi ≤ 2 = Indifference, FICi > 2 = Antagonism (European Committee for Antimicrobial Susceptibility Testing of the European Society of Clinical and Infectious 2000; Sanders et al. 1993; White et al. 1996).

### 2.5 Adherence to Plastic Surfaces via Crystal Violet Assay

The ability of NAC isolates to adhere on polystyrene surfaces were assessed as previously reported (Feoktistova et al. 2016). Yeast cells were grown for 24 h at 28°C, washed twice with sterile phosphate buffered saline (PBS), and resuspended at 37°C in RPMI 1640 supplemented with 10% fetal bovine serum (FBS) at 2.5 × 10^7^ cells. After 3 h of incubation at 37°C in six‐well polystyrene plates (Corning Incorporated, Corning, NY, USA), the medium was aspirated, and non‐adherent cells were removed with PBS. Adherent cells were fixed with 99% methanol (v/v) for 15 min, followed by staining with 0.02% crystal violet (v/v) for 20 min. After washing, 33% acetic acid (v/v) was added for 30 min, and the released crystal violet was quantified at 590 nm. The percentage of adherence was calculated using the following formula: [OD590 of the sample/OD590 of the control].

### 2.6 Biofilm Formation Assay

Biofilm formation was assessed using a previously described method (Finkel and Mitchell 2011). A yeast cell suspension (2.5 × 10^7^ cells/mL) was incubated in 96‐well microtiter plates (Corning, NY, USA) for either 3 h (early biofilm formation) or 24 h (pre‐formed biofilm) at 37°C. Fluconazole and MC1568, alone or in combination, were added at their respective MIC concentrations. After 24 h of incubation with the compounds, biofilm metabolic activity was evaluated using the 2,3‐bis‐(2‐methoxy‐4‐nitro‐5‐sulfophenyl)‐2*H*‐tetrazolium‐5‐carboxanilide (XTT)‐reduction assay (Ramage et al. 2001). Briefly, biofilm cells were washed with PBS and incubated with 0.5 mg/mL XTT and 1 μM menadione in PBS at 37°C for 2 h. A 500 μL sample was transferred from each well to a fresh 12‐well plate, and the colorimetric change from XTT reduction was measured at 490 nm. Biofilm cultures were prepared in triplicate, and each experiment was performed three times.

### 2.7 Infection Model in *G. mellonella*

For survival analyses, groups of *G. mellonella* ten larvae (250–320 mg/each) per *Candida* isolate were used, alongside two control groups. Larvae were pre‐incubated at 37°C and randomly assigned to experimental groups. Yeast cells (1 × 10^7^ cells/mL) were injected into the hemocoel through the last left proleg using a Hamilton syringe (701 N; 10 μL volume; 26 s needle; cone tip; Sigma‐Aldrich, Milan, Italy) (Smith and Casadevall 2021). After a 1‐h infection period, larvae were treated with Fluconazole or MC1568, alone or in combination, and incubated in Petri dishes at 37°C under standard aerobic conditions (Stringaro et al. 2022). All compounds were diluted in sterile water. Each of them was delivered once by an injection to the last right pro‐leg. A mock inoculation with PBS was performed in each experiment to monitor killing due to physical injury or infection by pathogenic contaminants. Survival was monitored at 24‐h intervals for 5 days. Larvae were considered dead when they displayed no movement in response to gentle prodding with a pipette tip. Two control groups were included: one without injections and another injected with PBS plus 0.01% Tween 20. Each experiment was repeated three times.

### 2.8 Statistical Analysis

Statistical differences between groups were analyzed using a Student's *t*‐test, *p*‐value of ≤ 0.05 was considered statistically significant. For survival rates, the log‐rank (Mantel‐Cox) test method was used. All analyses were performed using GraphPad Prism Software (version 8.0.1).

### 2.9 Determination of Cytotoxicity

Human commercial retinal epithelium cells (RPE‐1‐hTERT), kindly obtained from Dr Giulia Guarguaglini (CNR‐IBPM), were maintained in Dulbecco's Modified Eagle Medium‐F12 medium supplemented with 10% FBS, 1% glutamine and 1% of antibiotics. Cells were grown at 37°C in a humidified atmosphere, with 5% CO_2_. For experiments, 7000 cells were seeded in 200 μL of complete medium in each well of a 96‐well microtiter plate. After 24 h, 2.5, 5, and 10 µM of MC1568 was added in combination with 7.5, 15, and 30 µM of Fluconazole for 48 and 72 h in sextuplicate. The cytotoxic effect of drugs was assessed by added 0.5 mg/mL 3‐(4,5‐dimethylthiazol‐2‐yl)‐2,5‐diphenyltetrazolium bromide (MTT) (Sigma, St. Louis, MO, USA) for 4 h at 37°C and dissolved in 200 μL of isopropyl alcohol. Absorbance was measured at 570 nm (ELISA reader, DASIT) and cell viability was calculated as:

$$\text{Viability}=\left(\frac{\mathrm{OD}\;\mathrm{of}\;\mathrm{treated}\;\mathrm{cells}\;}{\mathrm{OD}\;\mathrm{of}\;\mathrm{control}\;\mathrm{cells}}\right)\times 100.$$

## 3 Results

### 3.1 Antimicrobial Activity of Clinically Approved HDACi and MC1568

In the first set of experiments, the antimicrobial activity of Fluconazole and of four HDACi (Vorinostat, Romidepsin, Tucidinostat, and MC1568) was assessed in 14 clinical isolates of *Candida* spp. at 24 and 48 h. The results are summarized in Tables 2 and 3. After 24 h of incubation, the MIC ranges were as follows: 0.125–64 μg/mL for Fluconazole, 0.25–8 μg/mL for Vorinostat, Romidepsin, and Tucidinostat, and 0.125–8 μg/mL for MC1568. After 48 h of incubation, the MIC ranges for Fluconazole and MC1568 remained unchanged, whereas the MIC values for the three clinically approved HDAC inhibitors increased significantly, reaching 2–8 μg/mL for Romidepsin and Vorinostat and 8 μg/mL for Tucidinostat. Between 24 and 48 h, the MIC_50_ (MIC 50%, the concentration of antimicrobial agent required to inhibit the growth of 50% of a tested microbial population) value for Fluconazole increased from 16 to 64 μg/mL, while for MC1568, it rose from 1 to 8 μg/mL, with approximately 60% of Fluconazole‐resistant strains showing susceptibility to MC1568. In contrast, the MIC_50_ values for the three approved HDACi remained stable at 8 μg/mL over the same period.

**Table 2:** Antimicrobial activity of Fluconazole and the tested HDACi against *Candida* spp. after 24 h of treatment.

*Candida* spp	MIC µg/mL, 24 h				
	FLC^a^	VOR^b^	ROM^c^	TUC^d^	MC1568
*C. guillermondii* 1511	16	4	8	> 8	0.125
*C. guillermondii* 1444	64	8	8	8	8
*C. lusitaniae* 301	0.125	8	8	8	1
*C. lusitaniae* 1112	64	8	8	8	2
*C. haemulonii* 1375	1	0.5	4	0.5	1
*C. haemulonii* 1293	64	0.25	0.25	0.25	0.25
*C. krusei* 45709	32	8	8	8	1
*C. krusei* 4495	32	8	8	8	2
*C. krusei* 1465	64	8	8	8	2
*C. glabrata* 43976	1	1	2	8	0.5
*C. glabrata* 1099	1	8	8	8	0.25
*C. glabrata complex* 23	64	8	8	8	0.5
*C. glabrata* DSY 562	0.5	8	8	8	8
*C. tropicalis* 14615	1	8	8	8	8
*MIC range*	0.125–64	0.25–8	0.25–8	0.25–8	0.125–8
*MIC*_*M*_	24.33	6.12	6.73	6.33	2.47
*MIC*_*50*_	16	8	8	8	2

^a^ FLC, Fluconazole.

^b^ VOR, Vorinostat.

^c^ ROM, Romidepsin.

^d^ TUC, Tucidinostat.

**Table 3:** Antimicrobial activity of Fluconazole and the tested HDACi against *Candida* spp. after 48 h of treatment.

*Candida* spp	MIC µg/mL, 48 h				
	FLC^a^	VOR^b^	ROM^c^	TUC^d^	MC1568
*C. guillermondii* 1511	16	4	> 8	> 8	0.125
*C. guillermondii* 1444	64	8	8	8	8
*C. lusitaniae* 301	0.125	8	8	8	8
*C. lusitaniae* 1112	64	8	8	8	8
*C. haemulonii* 1375	2	2	4	8	1
*C. haemulonii* 1293	64	8	8	8	0.25
*C. krusei* 45709	64	8	8	8	8
*C. krusei* 4495	64	8	8	8	8
*C. krusei* 1465	64	8	8	8	4
*C. glabrata* 43976	2	8	2	8	0.5
*C. glabrata* 1099	2	8	8	8	8
*C. glabrata complex* 23	64	8	8	8	0.5
*C. glabrata* DSY 562	0.5	8	8	8	8
*C. tropicalis* 14615	64	8	8	8	8
*MIC range*	0.125–64	2‐8	2‐8	8	0.125‐8
*MIC*_*M*_	38.18	7.28	7.28	8	4.45
*MIC*_*50*_	64	8	8	8	8

^a^ FLC, Fluconazole.

^b^ VOR, Vorinostat.

^c^ ROM, Romidepsin.

^d^ TUC, Tucidinostat.

### 3.2 Effects of Fluconazole in Combination With the Tested HDACi

The combination of antifungal agents with other compounds represents a potential strategy to enhance the efficacy of existing treatments. Tables 4 and 5 summarize the MIC values obtained for Fluconazole in combination with Vorinostat, Romidepsin, Tucidinostat, or MC1568 at 24 and 48 h in *Candida* spp. strains. The MIC was defined as the lowest concentrations of compounds that completely inhibited the growth of the organism as detected with the naked eye. *Candida* spp. isolates in which no one combination did not reach the minimum growth inhibition are not reported. The combination of Fluconazole with MC1568 displayed synergy in four tested strains, with FICi ranging from 0.09 to 0.19 after 48 h. The lowest FICi values were observed in *C. guillermondii* strains 1511 and 1444, *C. lusitaniae* strain 1112, and *C. tropicalis* strain 14515. Notably, in these strains, the MIC values for Fluconazole and MC1568 in combination were significantly lower (1/16–1/32 for Fluconazole and 1/8–1/16 for MC1568) than when each compound was used alone. The Fluconazole/Romidepsin combination exhibited synergy in three tested strains, with a FICi index ranging from 0.19 to 0.26 after 48 h. However, given the more promising results obtained with the Fluconazole/MC1568 combination, we decided to further investigate the antifungal properties of MC1568 in NAC species.

**Table 4:** Effect of combination of Fluconazole with HDACi on *Candida* spp. isolates after 24 h of treatment.

*Candida* spp.	FLC^a^/VOR^b^ combo, MICs (µg/mL)	FICi value (Effect)^c^	FLC/ROM^d^ combo, MICs (µg/mL)	FICi value (Effect)	FLC/TUC^e^ combo, MICs (µg/mL)	FICi value (Effect)	FLC/MC1568 combo, MICs (µg/mL)	FICi value (Effect)
*C. guillermondii* 1511	8/0.25	0.57 (Add)	8/0.25	0.54 (Add)	8/0.25	0.54 (Add)	1/1	0.19 (Syn)
*C. guillermondii* 1444	NI^f^	NI	4/1	0.19 (Syn)	32/0.25	0.53 (Add)	2/1	0.16 (Syn)
*C. lusitaniae* 1112	32/0.25	0.54 (Add)	2/1	0.16 (Syn)	NI	NI	0.5/1	0.13 (Syn)
*C. krusei* 45709	NI	NI	16/0.25	0.54 (Add)	NI	NI	16/0.25	0.37 (Syn)
*C. krusei* 4495	NI	NI	8/2	0.47 (Syn)	NI	NI	NI	NI
*C. tropicalis* 14615	NI	NI	0.5/0.5	0.56 (Add)	NI	NI	0.5/0.5	0.56 (Add)

^a^ FLC, Fluconazole.

^b^ VOR, Vorinostat.

^c^ Effect: Syn, synergy; Add, additivity.

^d^ ROM, Romidepsin.

^e^ TUC, Tucidinostat.

^f^ NI, no inhibition.

**Table 5:** Effect of combination of Fluconazole with HDACi on selected *Candida* spp. isolates after 48 h of treatment.

*Candida* spp.	FLC^a^/VOR^b^ combo, MICs (µg/mL)	FICi value (Effect)^c^	FLC/ROM^d^ combo, MICs (µg/mL)	FICi value (Effect)	FLC/TUC^e^ combo, MICs (µg/mL)	FICi value (Effect)	FLC/MC1568 combo, MICs (µg/mL)	FICi value (Effect)
*C. guillermondii* 1511	8/0.25	0.57 (Add)	8/0.25	0.54 (Add)	8/0.25	0.54 (Add)	1/1	0.19 (Syn)
*C. guillermondii* 1444	NI^f^	NI	4/1	0.19 (Syn)	NI	NI	4/1	0.16 (Syn)
*C. lusitaniae* 1112	NI	NI	4/1	0.19 (Syn)	NI	NI	2/1	0.15 (Syn)
*C. krusei* 45709	NI	NI	NI	NI	NI	NI	NI	NI
*C. krusei* 4495	NI	NI	NI	NI	NI	NI	NI	NI
*C. tropicalis* 14615	NI	NI	1/2	0.26 (Syn)	NI	NI	2/0.5	0.09 (Syn)

^a^ FLC, Fluconazole.

^b^ VOR, Vorinostat.

^c^ Effect: Syn, synergy; Add, additivity.

^d^ ROM, Romidepsin.

^e^ TUC, Tucidinostat.

^f^ NI, no inhibition.

### 3.3 Adherence of Selected *Candida* spp. Strains to Plastic Surfaces in the Presence of Fluconazole and MC1568, Alone or in Combination

**Figure 2:**
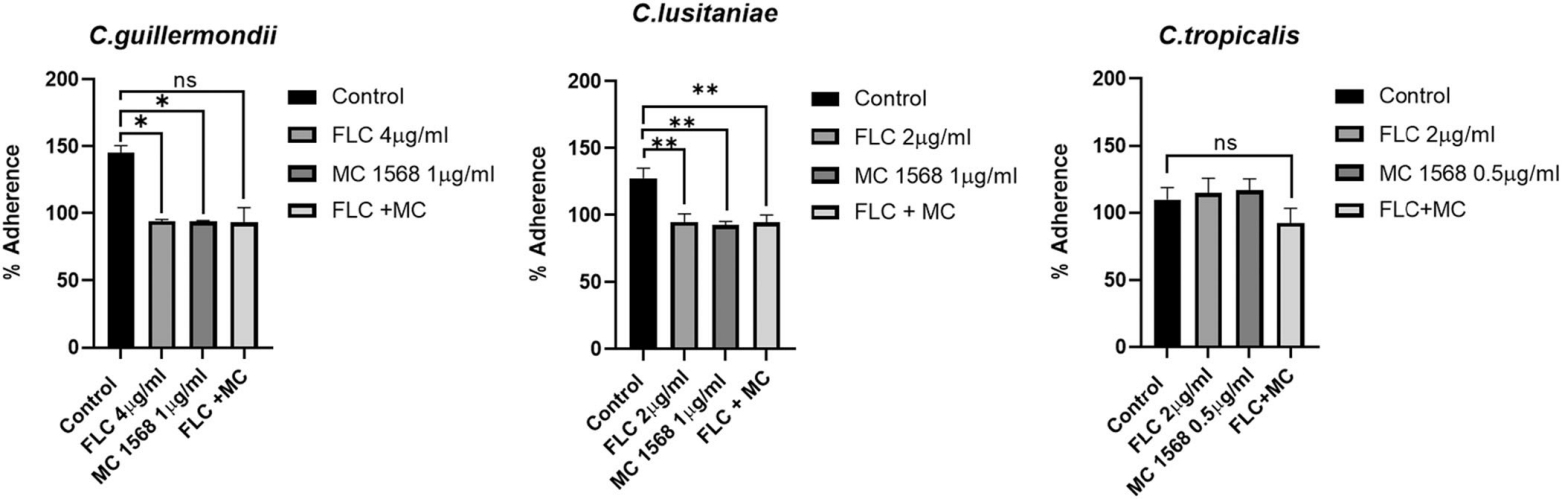
Histograms depicting adherence percentages on plastic surfaces in the presence of Fluconazole and MC1568, alone or in combination, in selected *Candida* spp. The two compounds were used at their MIC concentrations for combination experiments at 48 h. *p*‐values were obtained using a Student's *t*‐test (*p < 0.05, **p < 0.01, and ***p < 0.001) for three independent experiments.

Adherence to plastic surfaces is a critical virulence factor that facilitates infection and biofilm formation (Tronchin et al. 2008). The ability of *C. guillermondii, C. lusitaniae*, and *C. tropicalis* isolates to adhere to polystyrene in the presence of Fluconazole and MC1568, either alone or in combination, is shown in Figure 2.

All strains exhibited high adherence to polystyrene surfaces in the absence of treatment. When treated with Fluconazole or MC1568 at MIC concentrations (48 h), either alone or in combination, adherence was reduced by approximately 30% in *C. lusitaniae*, both when the compounds were used individually and in combination. In *C. guillermondii*, adherence decreased by ~40% when treated with Fluconazole or MC1568 alone, but the combination did not yield significant reductions. In contrast, *C. tropicalis* showed only a slight (~20%) but not significant decrease in adherence when treated with the Fluconazole/MC1568 combination, while neither compound alone significantly affected adherence (Figure 2).

### 3.4 Effect of Fluconazole and MC1568, Alone or in Combination, on Early and Mature Biofilms in Selected *Candida* spp. Strains

**Figure 3:**
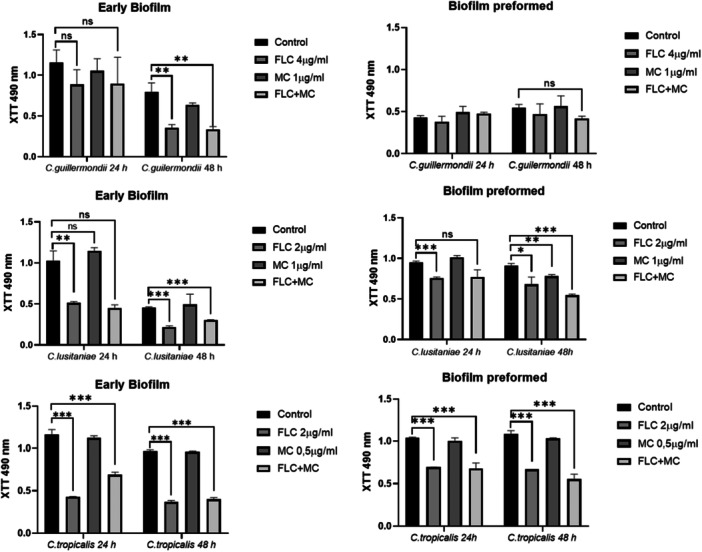
Histograms showing the inhibition of early and mature (pre‐formed) biofilms by Fluconazole and MC1568, alone or in combination, in selected *Candida* spp. after 24 and 48 h. The effects were measured using the XXT method. *p*‐values were obtained using a Student's *t*‐test (*p < 0.05, **p < 0.01, and ***p < 0.001) for three independent experiments.

The ability of Fluconazole and MC1568, individually or in combination, to inhibit biofilm formation was evaluated in *C. guillermondii, C. lusitaniae*, and *C. tropicalis* isolates. The compounds were added at two different time points: at the beginning of biofilm formation (early biofilm), and to a pre‐formed biofilm (mature biofilm). When added at the start of biofilm development, Fluconazole alone effectively inhibited biofilm formation at both 24 and 48 h across all three clinical isolates, though with species‐dependent variability. In contrast, MC1568 alone exhibited little or no inhibitory effect. The Fluconazole/MC1568 combination produced outcomes like Fluconazole alone, with varying degrees of biofilm inhibition and statistical significance: (*p* < 0.01) for *C. guillermondii* at 48 h, (*p* < 0.001) for *C. lusitaniae* at 48 h, and (*p* < 0.001) for *C. tropicalis* at 24 and 48 h (Figure 3).

When added to pre‐formed biofilms, Fluconazole alone was generally less effective than in early biofilm inhibition. MC1568 alone, as well as the Fluconazole/MC1568 combination, significantly inhibited pre‐formed biofilms in *C. lusitaniae* at 48 h (*p* < 0.01 for MC1568 alone, *p* < 0.001 for the combination), reducing biofilm formation by ~20% at 24 h and ~40% at 48 h (Figure 3). In *C. guillermondii*, neither Fluconazole, MC1568, nor their combination significantly inhibited biofilm growth. However, in *C. tropicalis*, Fluconazole alone had a strong inhibitory effect ( ~35% of inhibition) at both 24 and 48 h, while the combination further enhanced biofilm inhibition from 34% (24 h) to 49% (48 h) (Figure 3).

Thus, the Fluconazole/MC1568 combination exhibited a synergistic effect on mature biofilms of *C. lusitaniae* and *C. tropicalis*.

### 3.5 In Vivo Study on *G. mellonella* Larvae

*G. mellonella* larvae are broadly used as an invertebrate model to assess the virulence of microbial pathogens and the efficacy of antimicrobial agents (Cook and McArthur 2013; Giammarino et al. 2024). These larvae offer several advantages, including a simple nervous system, minimal ethical constraints, cost‐effectiveness, and the ability to survive at near‐human body temperatures ( ~ 37°C), which allows pathogen virulence factors to be expressed.

**Figure 4:**
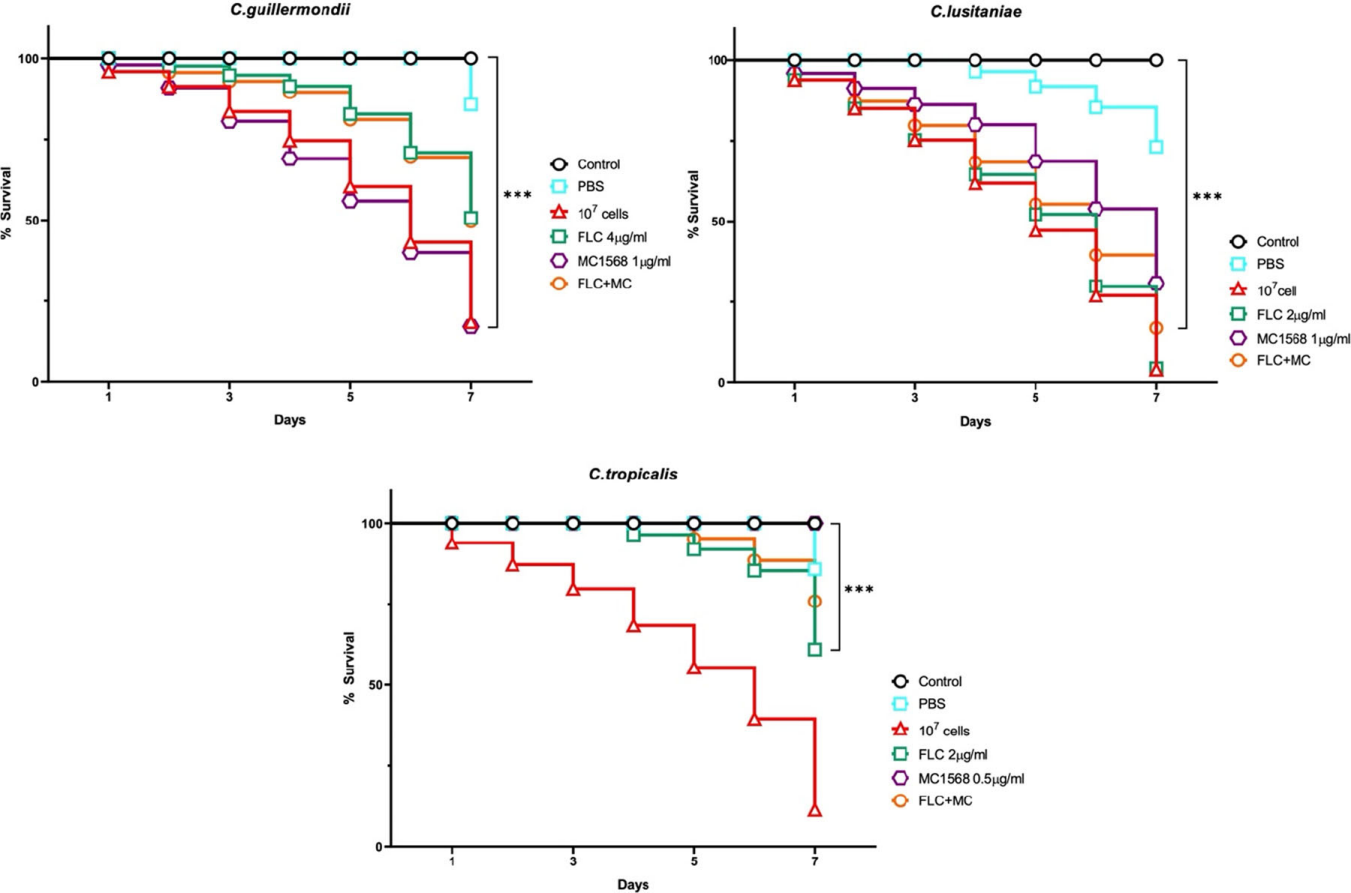
Survival percentages of *G. mellonella* larvae after infection with *C. guillermondii*, *C. lusitaniae*, and *C. tropicalis*, treated with Fluconazole and MC1568, alone or in combination, over seven days. For survival rates, the log‐rank (Mantel‐Cox) test method was used (***p < 0.001) for three independent experiments.

In this study, *G. mellonella* larvae were infected with *C. guillermondii*, *C. lusitaniae*, or *C. tropicalis* and treated with Fluconazole and MC1568, alone or in combination, at their MIC concentrations (48 h). Larval viability was monitored over 7 days (Figure 4). In *C. guillermondii* infected larvae, treatment with Fluconazole alone or the Fluconazole/MC1568 combination increased survival from 20% to 50% after 7 days. In *C. lusitaniae* infection, treatment with the combination led to a modest (~ 20%) improvement in survival. In *C. tropicalis* infection, the Fluconazole/MC1568 combination had a synergistic effect, increasing survival rates by 70% compared to the infected control.

### 3.6 Cytotoxicity of the Fluconazole/MC1568 Combination in Human Healthy Cells

To evaluate the cytotoxic effect of the Fluconazole/MC1568 combination in healthy human cells, human retinal pigment epithelium (RPE) cells were treated with MC1568 (2.5, 5, and 10 µM) in combination with Fluconazole (7.5, 15, and 30 µM, respectively) for 48 and 72 h. Cytotoxicity was assessed using the MTT assay.

**Figure 5:**
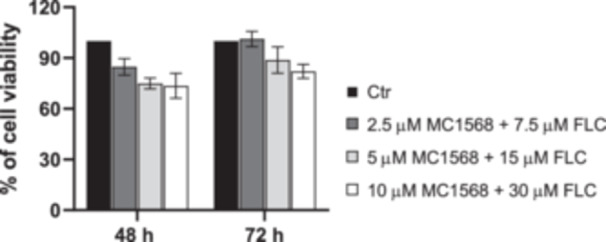
Percentages of reduction of RPE cell viability after co‐treatment with MC1568 (2.5, 5, and 10 μM) and Fluconazole (7.5, 15, and 30 μM) for 48 and 72 h, using the MTT method.

As shown in Figure 5, the three combinations exhibited minimal or no reduction in the viability of human RPE cells at both time points.

## 4 Discussion and Conclusions

Fungal infections pose a significant threat to human health, particularly in immunocompromised individuals. Invasive fungal infections are estimated to cause approximately 1.5 million deaths annually, with *Cryptococcus*, *Candida*, *Aspergillus*, and *Pneumocystis* species accounting for 90% of these fatalities. Notably, while *Candida* species typically inhabit epithelial surfaces as commensals, they can become opportunistic pathogens under specific conditions, such as immunosuppression and hospitalization (Brown et al. 2012). *Candida* species rank as the fourth leading cause of nosocomial bloodstream infections, with *C. albicans* being the most prevalent species (Pfaller and Diekema 2007). Estimates suggest that *C. albicans* is responsible for up to 400,000 infections annually, with mortality rates ranging from 46% to 75%. The situation is even more concerning for NAC infections, which account for 35%–65% of all cases of candidemia (Krcmery and Barnes 2002). These infections are more frequent in hospitalized cancer patients, particularly those with hematological malignancies and bone marrow transplant recipients (40%–70%), leading to high morbidity and mortality rates (Otto and Babady 2023). The prevalence of NAC infections has increased significantly, rising from 10% to 40% in 1990 to 35%–65% by 2000 (Krcmery and Barnes 2002).

Currently, the most used antifungal treatments include echinocandins (e.g., micafungin), amphotericin B, triazoles, and pyrimidines (Denning and Bromley 2015). However, the emergence of drug‐resistant strains highlights the urgent need for novel therapeutic approaches (Denning and Bromley 2015). Several studies suggest that targeting fungal HDACs could provide both direct antifungal effects and/or synergy with existing antifungal agents (de Oliveira et al. 2023; Mai et al. 2007; Pfaller et al. 2015; Simonetti et al. 2007; Smith and Edlind 2002).

In the present study, we evaluated the antifungal effects of three clinically approved HDAC inhibitors (Di Bello et al. 2022)—Vorinostat (a pan‐HDAC inhibitor), Romidepsin (a HDAC1‐3/10/11‐selective inhibitor), and Tucidinostat (a class I/HDAC10/11‐selective inhibitor)—along with the HDAC6/8‐selective inhibitor MC1568 (Mai et al. 2005; Noce et al. 2023; Panella et al. 2016). These compounds were tested both individually and in combination with the azole antifungal Fluconazole against various NAC species.

Initially, we assessed the antimicrobial activity of the three clinically approved HDACi and MC1568 against 14 clinical isolates of *Candida* spp. at 24 and 48 h, comparing their effects with Fluconazole. Our results demonstrated that the MIC range for MC1568 alone was lower than that of Fluconazole at both time points. Additionally, while the MIC range values for the three approved HDACi increased over time, their MIC_50_ values remained unchanged.

Next, we investigated the effects of combining Fluconazole with each of the three approved HDACi and MC1568 in the same *Candida* isolates using the checkerboard method (Orhan et al. 2005; White et al. 1996). Our findings revealed that the Fluconazole**/**MC1568 combination exhibited the most pronounced synergy, particularly in *C. guillermondii* strains 1511 and 1444, *C. lusitaniae* strain 1112, and *C. tropicalis* strain 14515. In these strains, the MIC values were significantly lower when the two drugs were used together compared to their individual administration at both 24 and 48 h. Given these promising results, we conducted further investigations focusing on MC1568, both as a monotherapy and in combination with Fluconazole, using the MIC concentrations established in the 48‐h combination experiments.

We first examined the effect of Fluconazole and MC1568, alone and in combination, on the adherence of *Candida* spp. to plastic surfaces (Figure 2). Our results demonstrated that both Fluconazole and MC1568, whether used individually or in combination, reduced adherence by approximately 30% in *C. lusitaniae* and 40% in *C. guillermondii*, while no significant change was observed in *C. tropicalis*.

Subsequently, we evaluated the effects of Fluconazole and MC1568, alone and in combination, on early and mature biofilm formation in *Candida* spp. (Figure 3). In early‐stage biofilms, Fluconazole consistently inhibited biofilm formation at both 24 and 48 h, except in *C. guillermondii* at 24 h. MC1568 alone showed minimal or no impact on early biofilm formation. However, when combined with Fluconazole, significant inhibition was observed in *C. guillermondii* and *C. lusitaniae* at 48 h, and in *C. tropicalis* at both 24 and 48 h. Regarding pre‐formed biofilms, Fluconazole alone exhibited reduced efficacy compared to its effect on early biofilms. MC1568 alone displayed a measurable effect only in *C. lusitaniae* at 48 h. Importantly, the Fluconazole/MC1568 combination demonstrated synergistic effects, particularly against *C. lusitaniae* at 48 h and *C. tropicalis* at both at 24 and 48 h. These findings are consistent with Rajasekharan et al. (2015), who reported that *C. tropicalis* biofilm formation was reduced in the presence of an HDACi combined with flavonoids (Rajasekharan et al. 2015).

Afterwards, we conducted an in vivo study using *G. mellonella* larvae infected with *Candida* spp., assessing survival rates over a 7‐day period following treatment with Fluconazole and MC1568, either alone or in combination (Figure 4). In larvae infected with *C. guillermondii*, treatment with Fluconazole alone or in combination with MC1568 improved survival rates from 20% to 50%. In *C. lusitaniae*‐infected larvae, none of the treatments significantly improved survival. However, in larvae infected with *C. tropicalis*, the Fluconazole/MC1568 combination exhibited a strong synergistic effect, increasing survival by 70%. Similar in vivo results have been observed with a carboline HDACi combined with Fluconazole in resistant strains of *C. albicans* (Li et al. 2021).

Finally, we tested three MC1568/Fluconazole combinations with increasing concentrations of both compounds (MC1568: 2.5, 5, and 10 μM; Fluconazole: 7.5, 15, and 30 μM) in human RPE cells at 48 and 72 h, observing low or no cytotoxicity (Figure 5). These findings underscore the potential of combining HDACi, particularly HDAC6/8‐selective inhibitors like MC1568, with azole antifungals as a promising strategy to combat NAC infections. Further research is warranted to identify optimal HDACi that can be effectively combined with existing antifungal agents to enhance treatment efficacy while minimizing cytotoxicity. In this regard, MC1568 appears to be a valid candidate, as it does not affect class I HDAC1‐3 isoforms (Noce et al. 2023; Panella et al. 2016) (Table 1) and thus exhibits minimal toxicity in human cells (Du et al. 2022; Naldi et al. 2009; Noce et al. 2023).

## Author Contributions

**Andrea Giammarino:** methodology; investigation; visualization; validation; software; data curation. **Chiara Lambona:** investigation; writing – review and editing; methodology; validation; data curation. **Alessia Raucci:** methodology; validation; visualization. **Giovanna Simonetti:** methodology; validation; visualization. **Salvatore Macrì:** methodology; validation; visualization. **Angela Luzzolino:** investigation; methodology; validation; visualization; data curation. **Daniela Trisciuoglio:** investigation; methodology; validation; visualization; data curation; software. **Sergio Valente:** methodology; validation; visualization; resources. **Gustavo Giusiano:** methodology; validation; visualization; data curation; supervision. **Letizia Angiolella:** conceptualization; investigation; funding acquisition; resources; writing – original draft; methodology; validation; visualization; data curation; formal analysis. **Antonello Mai:** conceptualization; investigation; funding acquisition; writing – review and editing; visualization; validation; methodology; formal analysis; data curation; supervision; resources.

## Ethics Statement

The authors have nothing report.

## Conflicts of Interest

None declared.

## Data Availability

All data generated or analyzed during this study are included in this published article.
